# Maternal adversity and peripheral proinflammatory cytokine concentrations in pregnancy

**DOI:** 10.1016/j.bbih.2026.101195

**Published:** 2026-02-12

**Authors:** Natalia Sifnugel, Sara B. Johnson, Raquel G. Hernandez, Cathrine Hoyo, Susan K. Murphy, Rachel Maguire, Jenna L. Riis

**Affiliations:** a NYU Grossman School of Medicine, Department of Emergency Medicine, 227 E 30th Street, New York, NY, 10016, USA; b Johns Hopkins University School of Medicine, Department of Pediatrics, 200 N. Wolfe St., Baltimore, MD, 21287, USA; c Johns Hopkins Bloomberg School of Public Health, 600 N. Wolfe St, Baltimore, MD, 21205, USA; d Johns Hopkins All Children's Hospital, 601 S. St, St. Petersburg, FL, 33701, USA; e North Carolina State University, Department of Biological Sciences, Campus Box 7633, Raleigh, NC, 27695-7633, USA; f Duke University, Department of Obstetrics and Gynecology, 701 W. Main Street, Suite 510, Durham, NC, 27701, USA; g University of Illinois Urbana-Champaign, College of Applied Health Sciences, Department of Health and Kinesiology, M/C 052, Urbana, IL, 61801, USA

**Keywords:** Pregnancy, Prenatal, Immune function, Cytokines, Adversity, Adverse childhood experiences

## Abstract

**Background:**

Psychosocial stressors such as adverse childhood experiences (ACEs) and financial hardship have been linked to inflammatory phenotypes. Few studies have investigated the role of childhood and current stressors on inflammatory marker concentrations in pregnancy, a period marked by shifts in immune activity. This study examined the associations among ACEs, financial stress, and concentrations of three cytokines: TNF-α, IL-6, and IL-8 in pregnant people. Additionally, we explored whether current financial stress amplified relations between ACEs and immune activity, and, in contrast, whether current social support served as a potential buffer.

**Methods:**

Pregnant people (n = 304) ages 18+ were enrolled from two prospective pregnancy cohorts in North Carolina and Florida between April 2018 and March 2020. Participant demographics, health behaviors, current health conditions, and documented medical and social histories were collected via survey and obstetric record abstraction. Venous blood samples were drawn and assayed for cytokines using commercially available multiplex kits. Associations between ACEs, financial stress, and cytokines were evaluated using multivariable tobit regression to account for left censoring of cytokine concentrations.

**Results:**

The mean age of the sample was 30 years and mean gestational age at blood collection was 26.8 weeks. Nearly three-fourths of participants had <2 ACEs, 61.5% were White, 38.5% were Black, and 24.0% were Hispanic. In both bivariate and multivariable analyses, IL-6 levels were decreased in the 2+ ACE group vs. the <2 ACE group (Exponentiated β = 0.39, p-value = 0.04). Financial stress significantly moderated the relationship between ACEs and IL-8 levels (Exponentiated β = 1.12, p-value = 0.04), revealing a negative association between financial stress and IL-8 levels in the <2 ACEs group and a positive association in the 2+ ACEs group.

**Conclusions:**

Our results suggest ACEs may have an immunosuppressive role in pregnant people. This relationship may be moderated by current social stressors. These results underscore the need for additional research to understand how life course experiences influence health and wellbeing for pregnant people, with implications for their children.

## Highlights

• Financial stress was linked to lower IL-6 levels in pregnancy in unadjusted models.
• 2+ ACEs was associated with lower IL-6 levels in pregnancy in adjusted models.
• Association between ACEs and IL-8 was moderated by current financial stress.
• IL-8 levels increased with increasing financial stress in the 2+ ACEs group.

## 1 Introduction

The immune system is diverse and expansive, providing ample opportunity for dysregulation (Liston et al., 2021). A common presentation of immune dysregulation is chronic inflammation, which poses serious risk to individual and population health as a mediator of cardiovascular disease, chronic obstructive pulmonary disease, mental health conditions and many other adverse health outcomes (Pahwa et al., 2025). The role of inflammation in common chronic disease pathology underscores the importance of reducing exposure to factors that disrupt immune balance, particularly in immune-compromised individuals such as pregnant people.

Both physiological and social factors influence immune activity. With respect to physiologic factors, pregnancy is marked by shifts in adaptive and innate immunity and cytokine expression to prevent rejection of the semi-allogenic fetus (Meyyazhagan et al., 2023). Perturbations to immune balance in this altered immune state may have harmful health consequences for both mother and child as proinflammatory bias in pregnancy is associated with poor outcomes such as preeclampsia and preterm birth (El-Shazly et al., 2004; Harmon et al., 2016).

Social experiences across the lifespan can also shape immune regulation. In early life, adverse childhood experiences (ACEs), such as abuse, neglect, or living with a household member with a substance use disorder, have been linked to proinflammatory immune profiles later in life. Specifically, previous studies have shown that adults who experienced abuse in childhood have higher levels of inflammatory markers such as C-reactive protein (CRP) (Baumeister et al., 2016; Danese et al., 2007; Kerr et al., 2021; Kuzminskaite et al., 2020; Pinto Pereira et al., 2019), IL-6, TNF-α (Hartwell et al., 2013; Miller and Cole, 2012), and soluble urokinase plasminogen activator receptor (suPAR) (Bourassa et al., 2021). In some studies, these relationships are independent of potential confounders such as current stress, health status, and health behavior (Danese et al., 2007). In other studies, the positive relationship between childhood adversity and inflammatory markers is attenuated after accounting for factors such as adult adiposity (Pinto Pereira et al., 2019), suggesting adversity in childhood may influence immune function indirectly. The notion of an indirect pathway is supported by research that finds that the positive relationship between early adversity and aggregate inflammatory marker levels (CRP, IL-6, fibrinogen, E-Selectin, and ICAM-1) is mediated by increased waist circumference, sedentary behavior, smoking, and higher urinary norepinephrine output (Hostinar et al., 2015).

One potential link between ACEs and immune function is social support. Several studies indicate that social support may buffer or amplify the relationship between ACEs and poor health outcomes; lower perceived social support is associated with increased risk for adverse health outcomes such as anxiety and depression, disorders associated with overactive immune systems, and substance use disorder (McCollum et al., 2024; Templeton et al., 2025). More directly, social support has been linked to lower levels of inflammation (Uchino et al., 2018). Collectively, these findings suggest social support may function as a protective factor for the effect of ACEs on maladaptive profiles of inflammation.

Limited resources and psychosocial stress related to material hardship have also been linked with changes in immune function. Lack of material resources was associated with elevated peripheral IL-6 (Surachman et al., 2023) in previous studies. Similarly, recession-related hardship increased linearly with both IL-6 and CRP in adults (Kirsch et al., 2023). In older populations, financial strain predicted higher TNF-α concentrations (Samuel et al., 2020), indicating the association between financial stress and immune function persists across the lifespan. Related but distinct from financial stress, socioeconomic status (e.g., income, education) has also been linked to inflammation such that individuals with lower socioeconomic status have greater levels of CRP and IL-6 (Fraga et al., 2015; Lam et al., 2021). These studies have spanned multiple Organisation for Economic Co-operation and Development (OECD) countries, further supporting the generalizability of the relationship between financial resources and immune function.

Although various studies have investigated the distinct roles of childhood adversity and adult financial stress in immune function, few have considered these associations among pregnant people. Since pregnancy is characterized by changes in inflammatory tone, associations in this population may differ from those observed in nonpregnant people. One study investigating maternal perceived stress found that high levels of stress were associated with elevated inflammation, as measured by peripheral CRP and IL-6 (Coussons-Read et al., 2007). Lymphocytes isolated from pregnant participants with high stress in this study produced more IL-6 upon stimulation compared to those from participants with low stress (Coussons-Read et al., 2007). Increased childhood disadvantage has also been associated with elevated circulating IL-6 in pregnancy, adjusting for age, race/ethnicity, parity, gestational hypertension, pre-eclampsia, and history of preterm birth (Miller et al., 2017). More recent research demonstrated that IL-6 concentrations in pregnant people were inversely associated socioeconomic status, but there was no significant relationship between IL-6 and childhood abuse (Finy and Christian, 2018). Additionally, the same study found that socioeconomic status was negatively associated with CRP, while childhood abuse were positively associated with CRP (Finy and Christian, 2018). Thus, the relationship between social experience and inflammation in pregnancy remains unclear.

Several gaps in our understanding of the relationships among adversity in childhood, adult financial stress, and proinflammatory activation in adulthood remain. To our knowledge, no studies to date have explicitly investigated the role of concurrent social stress, as measured by financial stress, in immune activity during pregnancy. Also, the impact of childhood adversity across immune markers is poorly understood as most of the current literature focuses solely on two indicators: CRP and IL-6. Centering on one cytokine limits our understanding of the relationships between social factors and the immune response because each cytokine has a unique role in inflammation. IL-6 signals for CRP synthesis by hepatocytes (Sproston and Ashworth, 2018), IL-8 recruits neutrophils to sites of trauma or infection, and TNF-α induces apoptosis (Obaji et al., 2025). Exclusively focusing on IL-6 and its downstream factors fails to capture individuals with expression patterns biased toward other known proinflammatory cytokines, which is likely to occur given that cytokine expression is regulated by genetic and environmental exposures and thus varies across individuals. To better characterize the relationship between adversity and inflammation, additional research is warranted.

To address these gaps in existing literature, the present study had four aims. First, we investigated the associations between maternal adversity in childhood (as measured by ACEs) and concentrations of three inflammatory markers in maternal blood (TNF-α, IL-6, and IL-8) during pregnancy. Second, we investigated the relationships between current maternal adversity (as measured by financial stress) and proinflammatory cytokine levels in pregnancy. Based on existing literature, we hypothesized that IL-6 and TNF-α would be positively associated with both ACEs and current financial stress. Few studies have measured IL-8 in this context; one prior study found no significant difference in cytokine concentrations between high and low maternal adversity groups (Miller et al., 2017). However, given its designation as an inflammatory cytokine and minimal existing research, we similarly hypothesized a positive association between IL-8 levels and both current and childhood adversity. Third, we investigated whether current maternal adversity modified the relationships between childhood adversity and proinflammatory cytokines. We hypothesized that the presence of current financial stress would exacerbate the associations between childhood adversity and proinflammatory cytokine levels. Finally, we considered the role of social support in modifying the relationships between ACEs and proinflammatory marker concentrations. We hypothesized that social support would attenuate the relationships between childhood adversity and cytokine concentrations.

## 2 Material and method

### 2.1 Study sample

This study used data from a sample of 577 pregnant people from two birth cohorts, Stress and Health in Pregnancy (SHIP) in North Carolina and Prospective Research on Early Determinants of Illness and Children's Health Trajectories (PREDICT) in Florida. Participants were enrolled from university-affiliated obstetric clinics between April 2018 and March 2020. Pregnant people were eligible if they were 18 years of age or older and planned to deliver at a study-affiliated hospital. For the PREDICT cohort, only English speakers were eligible, while for the SHIP cohort, both English and Spanish speakers could enroll. Individuals were ineligible if their fetus had a congenital or chromosome abnormality or if they had HIV, Hepatitis C, or Hepatitis B. Although included in the two cohorts, pregnant people carrying multiples (n = 24) were excluded from this analysis.

### 2.2 Study procedures

All study procedures were conducted at the affiliated obstetric clinic sites during the participants regularly scheduled prenatal appointments. Once enrolled, participants completed a survey that assessed maternal demographics and health behaviors, current health conditions, and documented medical and social histories. Additionally, when blood samples were drawn for clinical care, extra samples of venous blood were provided for study assays. Participants provided between one and three biospecimens on separate days across their pregnancy, depending on the needs of the care team, although only one sample per person was used in the current analyses (see 2.5.1). Participants’ obstetric history and delivery information were extracted from medical records. Mothers provided written informed consent. This study was approved by institutional review boards at both contributing sites.

### 2.3 Study measures

#### 2.3.1 Adverse childhood experiences

Adverse childhood experiences (ACEs) were ascertained by maternal self-report at enrollment using the 10-item measure developed by Felitti (Felitti et al., 1998). This measure captures experiences of abuse and household dysfunction including parental substance use, parental incarceration, and divorce before the age of 18. An ACE score (range: 0-10) was generated by summing the number of ACEs an individual experienced. Since the ACE score distribution was right-skewed (Mean = 1.16, Range = [0, 8]), scores were dichotomized as 2 or more vs. 0 or 1 ACEs for regression modeling following previous analyses with this sample (Sosnowski et al., 2023).

#### 2.3.2 Financial stress

Financial stress was measured using the six item Financial Stress Index, adapted from Essex et al., which assesses exposure to stressors such as difficulty paying bills and fear of losing home/job within the last three months (Essex et al., 2002). This measure captures the burden of financial stress across levels of material deprivation. Items include, “*How often have you had difficulty paying your bills*?” Responses were recorded on a Likert scale (0-4), where higher values indicate greater financial stress. A financial stress score (range: 0-24) was generated by summing ratings across stressors.

#### 2.3.3 Social support

Social support was measured using the eight item version of the Duke/UNC Functional Social Support Questionnaire (DUFSSQ) (Broadhead et al., 1988). The widely-used assessment captures multidimensional functional social support and has demonstrated construct validity and reliability (Broadhead et al., 1988). Participants are presented with a list of things that people do for them that may be helpful or supportive and they indicate that whether they get “as much as I would like” or “much less than I would like” for each item. Items include “visits with friends and relatives” and “love and affection.” Responses are scored on a 1-5 scale with higher values indicating greater social support. A social support score ranging from 8 to 40 was generated by summing responses across questions. Social support was dichotomized due to a right skewed distribution and to facilitate interpretation. Individuals with social support scores greater than 12.16, the sum of the mean score for each survey question, were classified as having high social support.

#### 2.3.4 Covariates

Covariates were selected based on our conceptual framework and previous research (Arnson et al., 2010; Baines and West, 2023; Khandaker et al., 2014; Milner and Beck, 2012). Multivariable models included: maternal age at delivery (years), gestational age at blood sample collection (weeks), maternal pre-pregnancy body mass index (BMI), tobacco use during pregnancy (yes/no), fetal sex (male/female), presence of a maternal cardiometabolic disorder (yes/no), and prior diagnosis of anxiety and/or depression (yes/no). Gestational age was defined based on weeks since last menstrual period. Presence of a cardiometabolic disorder was abstracted from the obstetric medical record and included diabetes, hypertension, obesity, and thyroid conditions. Prior diagnosis of anxiety and depression, gestational age (weeks), BMI, and fetal sex were also verified via abstracted obstetric records, while maternal age (years), and tobacco use (yes/no) were self-reported at enrollment.

### 2.4 Outcome assessment

#### 2.4.1 Blood collection, storage, and assays

During participants’ routine obstetric care, venous blood samples were collected. As allowed by their care team, a research-only specimen was collected at each blood draw between gestational ages 7.8-41.8 weeks. Of the total study sample (n = 577), 385 participants contributed blood samples. For the PREDICT cohort, plasma was separated from venous blood specimens using density-gradient centrifugation in EDTA anti-coagulant tubes. For the SHIP cohort, plasma was obtained using density-gradient centrifugation with Sigma-Aldrich Histopaque-1077 solution to prevent coagulation. All plasma samples were aliquoted and frozen at −80 °C until assayed.

#### 2.4.2 Cytokine levels

To investigate proinflammatory cytokine concentrations, TNF-α, IL-6, and IL-8 were measured in participant plasma samples. Assays were performed using commercially available multiplex kits (Millipore). Samples were run on thirteen different plates. Samples with low bead counts for any analyte were rerun on two additional plates, excess volume permitting. The upper and lower limits of quantification for each plate were determined based on the assay's standard curve. All analyte concentrations falling below the lower limit of quantification (LLOQ) were imputed as one-half the lower limit of quantification. No measures were above the upper limit of quantification.

### 2.5 Analytic methods

All analyses were conducted using R version 4.4.0 (R Core Team, 2024).

#### 2.5.1 Analytic sample

Among those in the study subsample who contributed venous blood samples (n = 385), 363 participants had data for all analytes. Cytokine data for these participants were joined with data from participant surveys and medical records, yielding a preliminary analytic sample of 345 participants.

Seventy-five percent of these participants had cytokine data from one blood sample and the remaining 25% (n = 87) had data from two blood samples over the course of the study. No participants in the merged exposure-outcome dataset had three collected samples. For this analysis, cytokine data from only one sample per participant was selected because there were no statistically significant differences in cytokine concentrations over time (paired Wilcoxson signed-rank test TNF-α: V = 1430, p = 0.08, IL-6: V = 251, p = 0.60, IL-8: V = 743, p = 0.82). The biospecimen used in the analysis was selected using the following scheme: if a participant had two blood samples, one from the first or third trimester and one from the second trimester, the sample from the second was kept. If a participant had one from the first trimester and one from the third trimester, the sample from the third was kept. If the participant had two samples from the second trimester, the one with the earliest gestational age was kept. No participants had multiple observations in the first or third trimester. This strategy was selected because most specimens were collected during later trimesters and the second trimester is characterized by lower levels of inflammatory markers as compared to earlier in pregnancy and close to delivery (Jarmund et al., 2021).

#### 2.5.2 Missing data

Of the 345 participants in the preliminary analytic sample, 41 (12.2%) were missing ACE scores. Since ACE category was our primary exposure, these individuals were removed from the sample, resulting in a final analytic sample of 304 individuals. Within the final analytic sample, missing data for covariates were as follows: Maternal Age at Delivery, 14 (4.6%); Financial Stress Score, 17 (5.6%); Maternal Pre-pregnancy BMI, 5 (1.6%); Ethnicity, 1 (0.3%); Social Support, 12 (3.9%), Fetal Sex, 6 (2.0%). All other covariate data (tobacco use during pregnancy, gestational age, cardiometabolic disorders, and prior diagnosis of anxiety/depression) were complete. Only cases with complete data were included in the regression analysis (n = 263-304).

#### 2.5.3 Analytic approach

To characterize the associations between maternal ACEs and cytokine levels and maternal current financial stress and cytokine levels we first evaluated bivariate associations using Spearman's rank correlation. Additionally, we used univariate general tobit regression models for cytokine concentrations with log-normal distributions. Separate models were run for each cytokine (TNF-α, IL-6, and IL-8). Cytokine concentrations were regressed on dichotomized ACE scores (<2, 2+) (n = 3 models) or continuous financial stress scores (n = 3 models), resulting in a total of six univariate models to assess unadjusted relations between ACEs, financial stress, and levels of the three cytokines. Then, we created separate multivariable tobit regression models for each cytokine, including dichotomized ACE and financial stress scores and covariates (maternal age at delivery, gestational age at blood sample collection, maternal pre-pregnancy BMI, tobacco use during pregnancy, fetal sex, maternal cardiometabolic disorder, and maternal prior diagnosis of anxiety and/or depression) to evaluate adjusted associations (n = 3 models). These methods were selected because cytokine data were left censored at their respective lower limits of measurement (IL-6: 3.27 pg/mL, IL-8: 3.23 pg/mL, TNF-α: 3.30 pg/mL; percent censored: TNF-α = 17.8, IL-6 = 68.1, IL-8 = 53.0) and not normally distributed (Ahmadi et al., 2021). Cytokine data were also Winsorized at the 99th percentile to reduce the influence of outliers (n = 3, 4, and 5 values Winsorized for IL-6, IL-8, and TNF-α, respectively).

To determine whether current financial stress exacerbated the relationships between ACEs and cytokine concentrations, we introduced an ACE category x financial stress score interaction term into multivariable tobit regression models (n = 3 models). Similarly, to determine whether social support buffered the relationship between ACEs and cytokine levels, we introduced an interaction term of ACE category x social support category into the tobit models (n = 3 models). Tobit regression models were conducted using the “tobit” function from the *AER version 1.2-13* package. Model fit was assessed by plotting residual values on fitted values.

#### 2.5.4 Sensitivity analyses

Some previous studies suggest that only individuals with the highest exposure to early adversity experience maladaptive shifts in immune function (Kuzminskaite et al., 2020). To investigate whether dichotomizing ACEs as 2+ versus 0 or 1 masked relationships between cytokines and ACE exposure at higher levels of exposure, we repeated the tobit analyses using ACE data with three levels of categorization (<2, 2-4, 4+ ACEs reported).

Additionally, to evaluate the potential effect of censored data on our findings, cytokine values, including those under the LLOQ, were split into three categories and their associations with ACEs was evaluated using ordinal regression predicting cytokine concentration group (e.g., low, medium, or high levels). TNF-α was categorized into tertiles based on the distribution (low: <4.85 pg/mL, medium: 4.85-8.25 pg/mL, high: *≥*8.25 pg/mL); IL-6 and IL-8 were categorized into tertiles, undetectable (below LLOQ), below median (below median pg/mL concentration excluding undetectable values), and above median (above median pg/mL concentration excluding undetectable values) because they had high frequencies of values below the LLOQ.

## 3 Results

### 3.1 Participant characteristics

Participant characteristics stratified by ACE category are reported in Table 1. Nearly three-fourths of participants reported fewer than two ACEs. The mean age of the sample was 30 years, and the mean gestational age at the time of blood collection was 26.8 weeks. The sample was 61.5% White, 38.5% Black, and 24.0% Hispanic. Mean pre-pregnancy BMI was 30.1 and 7.2% of participants smoked during pregnancy. Seventeen percent of participants had a cardiometabolic disorder and 5.3% had a prior diagnosis of anxiety and/or depression listed as a health complication in their obstetric records. Twenty-nine percent reported high social support. Of the 10 ACEs included in the screening tool, the most commonly reported ACEs in the sample were parental separation or divorce (26.6%), living with someone with a mental health disorder (15.1%), and living with someone with a substance abuse problem (14.5%). Participants in the analytic sample were not significantly different from remainder of the total sample with respect to ACE category, financial stress score, and all other covariates.

**Table 1:** Participant characteristics.

	<2 ACEs (*n* = 223)	2+ ACEs (*n* = 81)	Overall (*n* = 304)
Maternal Age (years)	30.0 (5.81)	30.1 (6.22)	30.0 (5.91)
Gestational Age (weeks)	26.7 (4.49)	26.9 (4.45)	26.8 (4.48)
Financial Stress Score	5.15 (5.39)	7.40 (5.74)	5.75 (5.56)
Maternal Pre-pregnancy Body Mass Index	30.6 (9.76)	28.8 (8.50)	30.1 (9.46)
Race			
Black	90 (40.4%)	27 (33.3%)	117 (38.5%)
White	133 (59.6%)	54 (66.7%)	187 (61.5%)
Hispanic Ethnicity	54 (24.2%)	19 (23.5%)	73 (24.0%)
Smoked while Pregnant	13 (5.8%)	9 (11.1%)	22 (7.2%)
Female Fetal Sex	98 (43.9%)	33 (40.7%)	131 (43.1%)
Cardiometabolic Comorbidity	43 (19.3%)	9 (11.1%)	52 (17.1%)
Prior Diagnosis of Anxiety or Depression	9 (4.0%)	7 (8.6%)	16 (5.3%)
Social Support			
High (*≥*2.16)	54 (24.2%)	34 (42.0%)	88 (28.9%)
Low (<12.16)	159 (71.3%)	45 (55.6%)	204 (67.1%)

Notes: Social support was measured using the Duke/UNC Functional Social Support Questionnaire (DUFSSQ). Social support scores were dichotomized at the sample mean score of 12.16 for analysis.

### 3.2 Cytokine concentrations

Descriptive statistics for TNF-α, IL-6, and IL-8 are reported in Table 2 including information about censoring. Censored cytokine concentrations (values below the LLOQ) were imputed as half the LLOQ for the calculation of summary statistics in Table 2 and the calculation of Spearman's correlation coefficients. IL-6 and IL-8 concentrations were highly correlated (ρ(302) = 0.87, *p* < 0.001), while TNF-α was weakly associated with both IL-6 (ρ (302) = 0.23, *p* < 0.001) and IL-8 (ρ (302) = 0.27, *p* < 0.001).

**Table 2:** Descriptive statistics for plasma cytokine concentrations assessed during pregnancy.

Cytokine	Mean pg/mL (Median, SD)	LLOQ (pg/mL)	Percent Censored (Below LLOQ)
TNF-α	8.66 (6.63, 19.18)	3.30	17.8%
IL-6	11.34 (1.6, 24.14)	3.27	68.1%
IL-8	12.09 (1.6, 22.65)	3.23	53.0%

### 3.3 Associations between childhood and current adversity and cytokine concentrations

Concentrations of IL-6 were inversely correlated with ACE scores, with higher ACE scores associated with lower concentrations of IL-6 (Table 3 and Supplemental Fig. 1). No statistically significant correlations were found between financial stress scores and cytokine concentrations.

**Table 3:** Correlations between cytokine concentrations and ACE and financial stress scores among a sample of pregnant people.

Cytokine	ACE Score Spearman's rho (*p*-value)	Financial Stress Score Spearman's rho, (*p*-value)
TNF-α	−0.02 (0.70)	0.07 (0.23)
IL-6	−0.11 (0.05)	−0.11 (0.07)
IL-8	−0.09 (0.13)	−0.02 (0.78)

The unadjusted and adjusted associations between the main adversity exposure variables (dichotomous ACE category and financial stress score) and cytokine concentrations from the tobit regression models are described below.

#### 3.3.1 TNF-α

Contrary to our hypothesis, TNF-α concentrations were not significantly associated with ACE category nor financial stress scores in unadjusted (Table 4) or adjusted models (Table 5). However, in sensitivity analyses using multivariable ordinal logistic regression, the odds of higher TNF-α levels increased by 5% per unit increase in financial stress score (OR = 1.05, p = 0.03) **(**Supplementary Table 2**).**

**Table 4:** Unadjusted associations between adverse childhood experiences (ACE) exposure category and current financial stress scores and cytokine concentrations among a sample of pregnant people.

Cytokine	Predictor	Exponentiated β	*p*-value
TNF-α	0-1 ACEs (ref)	-	
	2+ ACEs	0.99	0.89
	Financial Stress	1.01	0.44
IL-6	0-1 ACEs (ref)		
	2+ ACEs	0.40	0.02
	Financial Stress	0.93	0.04
IL-8	0-1 ACEs (ref)	-	
	2+ ACEs	0.61	0.06
	Financial Stress	0.98	0.42

Notes: Results generated from six univariate tobit regression models, one for each predictor (ACEs and Financial Stress) and outcome (TNF-α, IL-6, and IL-8) pair. ACEs are dichotomized as 0-1 ACEs and 2+ ACEs, and financial stress score is continuous.

**Table 5:** Adjusted associations between adverse childhood experiences (ACE) exposure category and current financial stress scores and cytokine concentrations among a sample of pregnant people.

Cytokine	Predictor	Exponentiated β	*p*-value
TNF-α	0-1 ACEs (ref)	-	
	2+ ACEs	1.01	0.88
	Financial Stress	1.01	0.16
IL-6	0-1 ACEs (ref)		
	2+ ACEs	0.39	0.04
	Financial Stress	0.95	0.15
IL-8	0-1 ACEs (ref)	-	
	2+ ACEs	0.60	0.08
	Financial Stress	0.99	0.80

Notes: Results generated from three multivariable tobit regression models, one for each cytokine as the outcome variable. ACEs are dichotomized as 0-1 ACEs and 2+ ACEs, and financial stress score is continuous. Multivariable model estimates are adjusted for maternal age at delivery, gestational age at blood sample draw, maternal pre-pregnancy BMI, tobacco use during pregnancy, fetal sex, presence of cardiometabolic disorders, and prior diagnosis of anxiety and/or depression.

#### 3.3.2 IL-6

Like bivariate correlation results, and contrary to our hypothesis, results from unadjusted tobit regression models showed that women reporting 2+ ACEs had, on average, lower IL-6 concentrations compared to women reporting 0-1 ACEs (Table 4). This difference in IL-6 levels across ACE category remained statistically significant after adjusting for financial stress, maternal age at delivery, gestational age at blood sample draw, maternal pre-pregnancy BMI, tobacco use during pregnancy, fetal sex, presence of cardiometabolic disorders, and prior diagnosis of anxiety and/or depression (Table 5). Results from adjusted models revealed that IL-6 concentrations were, on average, 61.0% lower in the 2+ ACEs group compared to the <2 ACEs group (Table 5).

In unadjusted, univariate tobit models, IL-6 concentrations decreased 7% per unit increase in financial stress score (Table 4), but this association was not statistically significant after adjusting for key covariates (Table 5).

The results from sensitivity analyses assessing these relationships using categorical cytokine concentrations and ordinal logistic regression were consistent with main findings reported here (Supplemental Table 2).

#### 3.3.3 IL-8

In unadjusted and adjusted tobit models, there were no statistically significant associations between IL-8 levels and financial stress scores. Contrary to our hypothesis, in all tobit models, women reporting 2+ ACEs showed lower IL-8 concentrations at the trend level as compared to those reporting <2 ACEs (Table 4, Table 5).

#### 3.3.4 Moderating role of financial stress

**Fig. 1:**
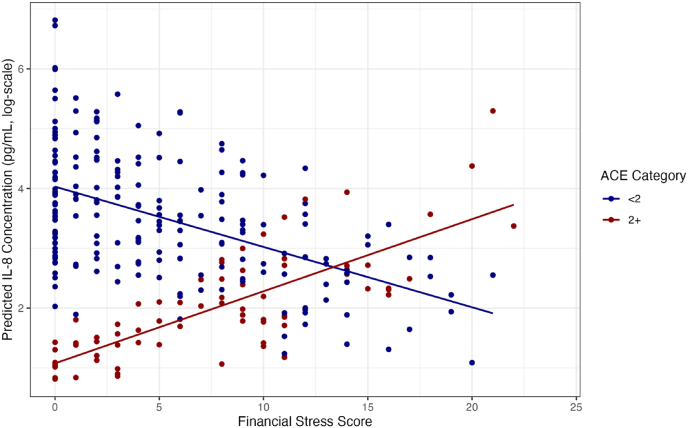
– Scatterplot of predicted log(IL-8 concentration) by financial stress across adverse childhood experiences (ACE) exposure categories.

In multivariable tobit models for IL-8 concentrations, including an interaction term for ACE category and financial stress, the main effect for financial stress was not significant, in line with previous results. Conversely, the main effect for ACE category (Exponentiated β = 0.26, p-value = 0.01) and the interaction term of ACE category and financial stress (Exponentiated β = 1.12, p-value = 0.04) were significant, suggesting financial stress modified the relationship between ACEs and IL-8 concentrations. Plotting predicted IL-8 concentrations versus financial stress score for the two ACEs groups illustrates a negative relationship between financial stress and IL-8 concentrations among low ACE individuals (<2 ACEs) and a positive relationship between financial stress and IL-8 concentrations among high ACE individuals (2+ ACEs) (Fig. 1). However, predicted IL-8 levels for high ACE, high financial stress individuals still fell below those for low ACE, low financial stress individuals. Results from sensitivity analyses were consistent with these findings. All other interaction effects tested between financial stress and ACE category on cytokine levels were not statistically significant.

#### 3.3.5 Moderating role of social support

In contrast to our hypotheses, there was no evidence that social support was a significant moderator of the relationship between childhood adversity and cytokine concentrations in multivariable models. Results from sensitivity analyses in which social support was treated continuously were consistent with results from the models that included dichotomized social support data.

## 4 Discussion

The aim of this study was to determine whether exposure to adversity, both during childhood (as measured by adverse childhood experiences, ACEs) and current (as measured by financial stress), is associated with levels of proinflammatory cytokines in pregnant people. Contrary to our hypotheses, we found that pregnant individuals who experienced two or more ACEs had lower levels of IL-6 compared to individuals with less than two ACE exposures. Similarly, IL-8 levels were lower in the 2+ ACE group than those in the lower ACE group, although this difference was only marginally significant. TNF-α concentrations did not differ by childhood or current adversity. We also did not find evidence of a buffering role of current social support on the relationship between childhood adversity and adult proinflammatory cytokine concentrations. However, in line with our hypothesis, financial stress modified the relationship between ACEs group and IL-8 concentrations in pregnancy such that the difference in IL-8 levels between individuals with high vs. low ACEs changed as financial stress score increased. Among participants reporting high ACEs, increased financial stress was associated with increased IL-8 concentrations, while the opposite pattern was observed among those reporting low ACEs.

These results suggest the need for further examination of the complex relationships among the timing of social experiences, immune function, and reproductive biology given equivocal results across studies and immune markers of interest. Finy and Christian found that both childhood abuse and current lower socioeconomic status were positively associated with IL-6 and CRP levels. However, adjusted analyses showed the relationship between childhood abuse and IL-6 was no longer significant (Finy and Christian, 2018). Consistent with Finy and Christian, Mitchell et al., found that childhood abuse and neglect were associated with elevated CRP in pregnancy (Mitchell et al., 2018). The persistent association between abuse and CRP supports a potential link between childhood adversity and future inflammation that we did not detect in these three cytokines.

Our results contradict those of Walsh et al. which found no association between child abuse and changes in IL-6 levels in pregnant adolescents (Walsh et al., 2016). Walsh et al., also showed that IL-6 levels were elevated in participants with high abuse and high depression compared to those with high abuse and low depression, suggesting that individuals with increased exposure to childhood adversity may have greater susceptibility to immune changes via psychosocial moderators during pregnancy. Results from Walsh et al. may differ from ours since their sample included adolescents exclusively and individuals with depression. Depression is associated with increased peripheral inflammatory biomarkers (Miller et al., 2009), indicating that a sample of both non-depressed and depressed participants may yield a wider distribution of cytokine concentrations levels than a sample containing only non-depressed participants. No association was observed for CRP in their study, highlighting the complex and inconsistent nature of these relationships across the literature.

The results of this study should be considered in light of several limitations. First, the detection capability of the Millipore assay used to measure cytokine concentrations is narrow. Many individuals in the study had cytokine levels below the assay's detectable range such that 18% to 68% of observations were censored depending on the cytokine. We sought to address this limitation in two ways. First, we used tobit regression which is the most appropriate approach to model censored biomarker data (Ahmadi et al., 2021) and has been used with highly censored data (Hahn et al., 2019; Metayer et al., 2013; Peetsalu et al., 2022; Vogelaar et al., 2017). Second, we conducted a sensitivity analysis where cytokine concentrations were binned into three categories and associations were explored using ordinal logistic regression which has more relaxed assumptions compared to the tobit model as it relies on order not magnitude in the outcome. For cytokines with the highest levels of censoring, IL-6 and IL-8, these groups were undetectable, below the median of detectable values, and above the median of detectable values, allowing for comparison between values above and below the limits of measurement. The results of these analyses were consistent with the findings from the tobit models. The consistency of the results across analytic approach supports the robustness of our findings. While the ranges of cytokine concentrations found in our study are consistent with other published reports among pregnant people (Cemgil Arikan et al., 2012; Curry et al., 2008; Dibble et al., 2014), it is important to note that our biospecimen samples were not assayed in duplicate, which may have provided more information and reduced the rate of censoring, an important consideration for future research.

Another limitation was the right skew in the ACE score distribution with 73.4% of participants reporting less than two ACEs. This posed a challenge since similar studies have found altered proinflammatory cytokine levels only among participants on the higher end of the childhood adversity spectrum (Kuzminskaite et al., 2020). To explore the potential masking effect of dichotomizing ACEs, we conducted a sensitivity analysis where ACEs were split into three categories which revealed similar relationships to our main analyses. A third limitation is that both ACE and financial stress scores were lower in our sample compared to other pregnant samples (Fields et al., 2023; Taylor et al., 2021), which may reflect differences in our sample compared to others, or measurement biases such as recall bias or under-reporting due to stigma or shame. Fourth, the data may not be representative of the participants’ full range of cytokine expression across pregnancy because only one measure per participant was utilized. However, in the subset of participants with multiple measures, we did not observe statistically significant differences in cytokine concentrations across gestational age. Fifth, mental health disorders were abstracted from the medical records which may underestimate the true prevalence. Sixth, contributing clinics were in predominantly White, Black, and/or Hispanic/Latino communities, restricting our sample to these racial and ethnic groups and limiting generalizability. Lastly, dropping observations with missing data in regression analysis may have introduced selection biases.

Despite these limitations, our results show that childhood adversity may be associated with immune changes in a non-clinical sample of pregnant people. The mechanism by which adversity modulates immune activity hinges on their mutual interactions with the neuroendocrine system (Johnson et al., 2013). Adversity induces stress, which activates the hypothalamic-pituitary-adrenal (HPA) axis, triggering the release of glucocorticoids such as cortisol. Cortisol functions in a negative-feedback manner, inhibiting further signaling of the hypothalamus and pituitary gland and suppressing proinflammatory activity in immune cells to divert energy toward a fight-ready physiological state (Webster et al., 2002). Hyperactivation of this pathway via persistent exposure to adversity may disrupt its functioning (McEwen, 2000).

In this study, we observed that participants with higher childhood adversity had lower IL-6 and IL-8 levels. Differences in TNF-α were not statistically significant and this difference may be explained by the stimulatory role of TNF-α in IL-6 and IL-8 expression (Pease and Sabroe, 2002; Wolf et al., 2014), which suggests peak concentration of TNF-α may precede those of IL-6 and IL-8. Additionally, these differences may be explained in part by the distinct shifts in cytokine expression patterns during pregnancy (Spence et al., 2021). A biological explanation for decreased IL-6 and IL-8 expression is immunosuppression via excess glucocorticoids (Rhen and Cidlowski, 2005). Specifically, participants with higher adversity likely experienced chronic stress in childhood, increasing the frequency of cortisol release, inhibiting the synthesis of inflammatory cytokines by immune cells. This immunosuppressed phenotype in individuals who experienced adversity may be exacerbated during pregnancy since cortisol concentration rises across trimesters (Graham et al., 2019). However, this hypothesized biological mechanism is less supported in the literature than that which suggests childhood adversity induces chronic, low levels of inflammation via cortisol desensitization (Danese et al., 2007; Epstein et al., 2021). In our study, individuals with both current and vestigial exposures to stress (high ACE scores and high financial stress) had elevated predicted IL-8 levels as compared to those with either current or vestigial stress alone. This suggests that recent activation of glucocorticoid signaling via financial stress may promote inflammation only among those who are prone to immune dysregulation, i.e., those with higher ACEs exposure. It is important to note, however, that those with both high ACEs and high financial stress still had lower predicted IL-8 concentrations compared to those with low ACEs and low financial stress. Our results coupled with other research which indicates that markers such as IL-6 can have pro- or anti-inflammatory properties at different stages of pregnancy (Pandey et al., 2017) highlights the need for more comprehensive assessments of immune activity in this population to define the clinical significance of varying cytokine concentrations.

## 5 Conclusion

This analysis adds to the breadth of literature that demonstrates how childhood adversity may have deleterious effects on health across the life course and potentially across generations (Christiaens et al., 2015; Ciciolla et al., 2021; Miller et al., 2021; Shamblaw et al., 2021; Sosnowski et al., 2023). These results underscore the need for additional study to understand how life course experience of parents impacts health and wellbeing in both pregnant people and their children.

## CRediT authorship contribution statement

**Natalia Sifnugel:** Formal analysis, Writing – original draft. **Sara B. Johnson:** Conceptualization, Data curation, Funding acquisition, Investigation, Supervision, Writing – review & editing. **Raquel G. Hernandez:** Writing – review & editing. **Cathrine Hoyo:** Conceptualization, Writing – review & editing. **Susan K. Murphy:** Writing – review & editing. **Rachel Maguire:** Writing – review & editing. **Jenna L. Riis:** Methodology, Supervision, Writing – review & editing.

## Funding sources

This work was supported by NIH/NIMHD R01MD011746 and by the All Children's Hospital Foundation.

## Declaration of competing interest

The authors declare that they have no known competing financial interests or personal relationships that could have appeared to influence the work reported in this paper.

## Data Availability

The data that has been used is confidential.

## References

[bib1] Ahmadi H., Granger D.A., Hamilton K.R., Blair C., Riis J.L. (2021). Censored data considerations and analytical approaches for salivary bioscience data. Psychoneuroendocrinology.

[bib2] Arnson Y., Shoenfeld Y., Amital H. (2010). Effects of tobacco smoke on immunity, inflammation and autoimmunity. J. Autoimmun..

[bib3] Baines K.J., West R.C. (2023). Sex differences in innate and adaptive immunity impact fetal, placental, and maternal health. Biol. Reprod..

[bib4] Baumeister D., Akhtar R., Ciufolini S., Pariante C.M., Mondelli V. (2016). Childhood trauma and adulthood inflammation: a meta-analysis of peripheral C-reactive protein, interleukin-6 and tumour necrosis factor-α. Mol. Psychiatr..

[bib5] Bourassa K.J., Rasmussen L.J.H., Danese A., Eugen-Olsen J., Harrington H., Houts R., Poulton R., Ramrakha S., Sugden K., Williams B., Moffitt T.E., Caspi A. (2021). Linking stressful life events and chronic inflammation using suPAR (soluble urokinase plasminogen activator receptor). Brain Behav. Immun..

[bib6] Broadhead W.E., Gehlbach S.H., De Gruy F.V., Kaplan B.H. (1988). The Duke/UNC functional social support questionnaire: measurement of social support in family medicine patients. Med. Care.

[bib7] Cemgil Arikan D., Aral M., Coskun A., Ozer A. (2012). Plasma IL-4, IL-8, IL-12, interferon-γ and CRP levels in pregnant women with preeclampsia, and their relation with severity of disease and fetal birth weight. J. Matern. Fetal Neonatal Med..

[bib8] Christiaens I., Hegadoren K., Olson D.M. (2015). Adverse childhood experiences are associated with spontaneous preterm birth: a case–control study. BMC Med..

[bib9] Ciciolla L., Shreffler K.M., Tiemeyer S. (2021). Maternal childhood adversity as a risk for perinatal complications and NICU hospitalization. J. Pediatr. Psychol..

[bib10] Coussons-Read M.E., Okun M.L., Nettles C.D. (2007). Psychosocial stress increases inflammatory markers and alters cytokine production across pregnancy. Brain Behav. Immun..

[bib11] Curry A.E., Vogel I., Skogstrand K., Drews C., Schendel D.E., Flanders W.D., Hougaard D.M., Thorsen P. (2008). Maternal plasma cytokines in early- and mid-gestation of normal human pregnancy and their association with maternal factors. J. Reprod. Immunol..

[bib12] Danese A., Pariante C.M., Caspi A., Taylor A., Poulton R. (2007). Childhood maltreatment predicts adult inflammation in a life-course study. Proc. Natl. Acad. Sci..

[bib13] Dibble S., Andersen A., Lassen M.R., Cunanan J., Hoppensteadt D., Fareed J. (2014). Inflammatory and procoagulant cytokine levels during pregnancy as predictors of adverse obstetrical complications. Clin. Appl. Thromb. Off. J. Int. Acad. Clin. Appl. Thromb..

[bib14] El‐Shazly S., Makhseed M., Azizieh F., Raghupathy R. (2004). Increased expression of pro‐inflammatory cytokines in placentas of women undergoing spontaneous preterm delivery or premature rupture of membranes. Am. J. Reprod. Immunol..

[bib15] Epstein C.M., Houfek J.F., Rice M.J., Weiss S.J. (2021). Integrative review of early life adversity and cortisol regulation in pregnancy. J. Obstet. Gynecol. Neonatal Nurs..

[bib16] Essex M.J., Klein M.H., Cho E., Kalin N.H. (2002). Maternal stress beginning in infancy may sensitize children to later stress exposure: effects on cortisol and behavior. Biol. Psychiatry.

[bib17] Felitti V.J., Anda R.F., Nordenberg D., Williamson D.F., Spitz A.M., Edwards V., Koss M.P., Marks J.S. (1998). Relationship of childhood abuse and household dysfunction to many of the leading causes of death in adults. Am. J. Prev. Med..

[bib18] Fields K., Ciciolla L., Addante S., Erato G., Quigley A., Mullins-Sweatt S.N., Shreffler K.M. (2023). Maternal adverse childhood experiences and perceived stress during pregnancy: the role of personality. J. Child Adolesc. Trauma.

[bib19] Finy M.S., Christian L.M. (2018). Pathways linking childhood abuse history and current socioeconomic status to inflammation during pregnancy. Brain Behav. Immun..

[bib20] Fraga S., Marques-Vidal P., Vollenweider P., Waeber G., Guessous I., Paccaud F., Barros H., Stringhini S. (2015). Association of socioeconomic status with inflammatory markers: a two cohort comparison. Prev. Med..

[bib21] Graham A.M., Rasmussen J.M., Entringer S., Ben Ward E., Rudolph M.D., Gilmore J.H., Styner M., Wadhwa P.D., Fair D.A., Buss C. (2019). Maternal cortisol concentrations during pregnancy and sex specific associations with neonatal amygdala connectivity and emerging internalizing behaviors. Biol. Psychiatry.

[bib22] Hahn J., Gold D.R., Coull B.A., McCormick M.C., Finn P.W., Perkins D.L., Rich-Edwards J.W., Rifas Shiman S.L., Oken E., Kubzansky L.D. (2019). Prenatal maternal depression and neonatal immune responses. Psychosom. Med..

[bib23] Harmon A.C., Cornelius D.C., Amaral L.M., Faulkner J.L., Cunningham M.W., Wallace K., LaMarca B. (2016). The role of inflammation in the pathology of preeclampsia. Clin. Sci..

[bib24] Hartwell K.J., Moran-Santa Maria M.M., Twal W.O., Shaftman S., DeSantis S.M., McRae-Clark A.L., Brady K.T. (2013). Association of elevated cytokines with childhood adversity in a sample of healthy adults. J. Psychiatr. Res..

[bib25] Hostinar C.E., Lachman M.E., Mroczek D.K., Seeman T.E., Miller G.E. (2015). Additive contributions of childhood adversity and recent stressors to inflammation at midlife: findings from the MIDUS study. Dev. Psychol..

[bib26] Jarmund A.H., Giskeødegård G.F., Ryssdal M., Steinkjer B., Stokkeland L.M.T., Madssen T.S., Stafne S.N., Stridsklev S., Moholdt T., Heimstad R., Vanky E., Iversen A.-C. (2021). Cytokine patterns in maternal serum from first trimester to term and beyond. Front. Immunol..

[bib27] Johnson S.B., Riley A.W., Granger D.A., Riis J. (2013). The science of early life toxic stress for pediatric practice and advocacy. Pediatrics.

[bib28] Kerr D.M., McDonald J., Minnis H. (2021). The association of child maltreatment and systemic inflammation in adulthood: a systematic review. PLoS One.

[bib29] Khandaker G.M., Pearson R.M., Zammit S., Lewis G., Jones P.B. (2014). Association of serum interleukin 6 and C-Reactive protein in childhood with depression and psychosis in young adult life: a population-based longitudinal study. JAMA Psychiatry.

[bib30] Kirsch J.A., Coe C., Ryff C.D. (2023). Racial and educational disparities in cumulative exposure to hardships of the 2008 great recession and inflammation. Psychosom. Med..

[bib31] Kuzminskaite E., Vinkers C.H., Elzinga B.M., Wardenaar K.J., Giltay E.J., Penninx B.W.J.H. (2020). Childhood trauma and dysregulation of multiple biological stress systems in adulthood: results from the Netherlands study of depression and anxiety (NESDA). Psychoneuroendocrinology.

[bib32] Lam P.H., Chiang J.J., Chen E., Miller G.E. (2021). Race, socioeconomic status, and low-grade inflammatory biomarkers across the lifecourse: a pooled analysis of seven studies. Psychoneuroendocrinology.

[bib33] Liston A., Humblet-Baron S., Duffy D., Goris A. (2021). Human immune diversity: from evolution to modernity. Nat. Immunol..

[bib34] McCollum D.C., Teeters J.B., Moskal K.R., Woodward M.J. (2024). Does social support moderate the association between adverse childhood experiences and substance-related problems?. Subst. Use Misuse.

[bib35] McEwen B. (2000). Allostasis and allostatic load implications for neuropsychopharmacology. Neuropsychopharmacology.

[bib36] Metayer C., Colt J.S., Buffler P.A., Reed H.D., Selvin S., Crouse V., Ward M.H. (2013). Exposure to herbicides in house dust and risk of childhood acute lymphocytic leukemia. J. Expo. Sci. Environ. Epidemiol..

[bib37] Meyyazhagan A., Kuchi Bhotla H., Pappuswamy M., Tsibizova V., Al Qasem M., Di Renzo G.C. (2023). Cytokine see‐saw across pregnancy, its related complexities and consequences. Int. J. Gynecol. Obstet..

[bib38] Miller A.H., Maletic V., Raison C.L. (2009). Inflammation and its discontents: the role of cytokines in the pathophysiology of major depression. Biol. Psychiatry.

[bib39] Miller E.S., Fleming O., Ekpe E.E., Grobman W.A., Heard-Garris N. (2021). Association between adverse childhood experiences and adverse pregnancy outcomes. Obstet. Gynecol..

[bib40] Miller G.E., Cole S.W. (2012). Clustering of depression and inflammation in adolescents previously exposed to childhood adversity. Biol. Psychiatry.

[bib41] Miller G.E., Culhane J., Grobman W., Simhan H., Williamson D.E., Adam E.K., Buss C., Entringer S., Kim K.-Y., Felipe Garcia-Espana J., Keenan-Devlin L., McDade T.W., Wadhwa P.D., Borders A. (2017). Mothers' childhood hardship forecasts adverse pregnancy outcomes: role of inflammatory, lifestyle, and psychosocial pathways. Brain Behav. Immun..

[bib42] Milner J.J., Beck M.A. (2012). The impact of obesity on the immune response to infection. Proc. Nutr. Soc..

[bib43] Mitchell A.M., Porter K., Christian L.M. (2018). Examination of the role of obesity in the association between childhood trauma and inflammation during pregnancy. Health Psychol..

[bib44] Obaji M.U., Oli A.N., Ugwu M.C. (2025). The roles of IL-6, IL-8, and TNF-α in pediatric immune defense and infection severity. OBM Genet..

[bib45] Pahwa R., Goyal A., Jialal I. (2025). StatPearls.

[bib46] Pandey M., Chauhan M., Awasthi S. (2017). Interplay of cytokines in preterm birth. Indian J. Med. Res..

[bib47] Pease J.E., Sabroe I. (2002). The role of Interleukin-8 and its receptors in inflammatory lung disease. Am. J. Respir. Med..

[bib48] Peetsalu K., Niine T., Loch M., Dorbek-Kolin E., Tummeleht L., Orro T. (2022). Effect of colostrum on the acute-phase response in neonatal dairy calves. J. Dairy Sci..

[bib49] Pinto Pereira S.M., Stein Merkin S., Seeman T., Power C. (2019). Understanding associations of early-life adversities with mid-life inflammatory profiles: evidence from the UK and USA. Brain Behav. Immun..

[bib50] R Core Team (2024).

[bib51] Rhen T., Cidlowski J.A. (2005). Antiinflammatory action of glucocorticoids — new mechanisms for old drugs. N. Engl. J. Med..

[bib53] Samuel L., Szanton S.L., Fedarko N.S., Simonsick E.M. (2020). Leveraging naturally occurring variation in financial stress to examine associations with inflammatory burden among older adults. J. Epidemiol. Community Health.

[bib54] Shamblaw A.L., Sommer J.L., Reynolds K., Mota N., Afifi T.O., El-Gabalawy R. (2021). Pregnancy and obstetric complications in women with a history of childhood maltreatment: results from a nationally representative sample. Gen. Hosp. Psychiatry.

[bib55] Sosnowski D.W., Ellison-Barnes A., Kaufman J., Hoyo C., Murphy S.K., Hernandez R.G., Marchesoni J., Klein L.M., Johnson S.B. (2023). Financial stress as a mediator of the association between maternal childhood adversity and infant birth weight, gestational age, and NICU admission. BMC Public Health.

[bib56] Spence T., Allsopp P.J., Yeates A.J., Mulhern M.S., Strain J.J., McSorley E.M. (2021). Maternal serum cytokine concentrations in healthy pregnancy and preeclampsia. J. Pregnancy.

[bib57] Sproston N.R., Ashworth J.J. (2018). Role of C-Reactive protein at sites of inflammation and infection. Front. Immunol..

[bib58] Surachman A., Tucker-Seeley R., Almeida D.M. (2023). The association between material-psychological-behavioral framework of financial hardship and markers of inflammation: a cross-sectional study of the midlife in the united states (MIDUS) refresher cohort. BMC Public Health.

[bib59] Taylor K., Compton S., Kolenic G.E., Scott J., Becker N., Dalton V.K., Moniz M.H. (2021). Financial hardship among pregnant and postpartum women in the United States, 2013 to 2018. JAMA Netw. Open.

[bib60] Templeton J.M., Dixon W.E., Williams S., Morelen D., Driggers-Jones L., Robertson C. (2025). The mediating role of social support on the link between adverse childhood experiences and adult mental health. J. Exp. Child Psychol..

[bib61] Uchino B.N., Trettevik R., Kent de Grey R.G., Cronan S., Hogan J., Baucom B.R.W. (2018). Social support, social integration, and inflammatory cytokines: a meta-analysis. Health Psychol..

[bib62] Vogelaar L., de Haar C., Aerts B.R., Peppelenbosch M.P., Timman R., Hanssen B.E., van der Woude C.J. (2017). Fatigue in patients with inflammatory bowel disease is associated with distinct differences in immune parameters. Clin. Exp. Gastroenterol..

[bib63] Walsh K., Basu A., Werner E., Lee S., Feng T., Osborne L.M., Rainford A., Gilchrist M., Monk C. (2016). Associations among child abuse, depression, and Interleukin-6 in pregnant adolescents: paradoxical findings. Psychosom. Med..

[bib64] Webster J.I., Tonelli L., Sternberg E.M. (2002). Neuroendocrine regulation of immunity. Annu. Rev. Immunol..

[bib65] Wolf J., Rose-John S., Garbers C. (2014). Interleukin-6 and its receptors: a highly regulated and dynamic system. Cytokine.

