# Supplementary material for: Maternal adversity and peripheral proinflammatory cytokine concentrations in pregnancy
Source: Brain Behav Immun Health. 2026 Feb 12;53:101195. doi: 10.1016/j.bbih.2026.101195 (PMC12924726; doi:10.1016/j.bbih.2026.101195)
Supplement: Multimedia component 1 [file mmc1.docx]

Supplemental Tables and Figures

**Supplemental Figure 1 – Scatterplot of IL-6 concentrations by number of adverse childhood experiences (ACEs) among pregnant people.**

**
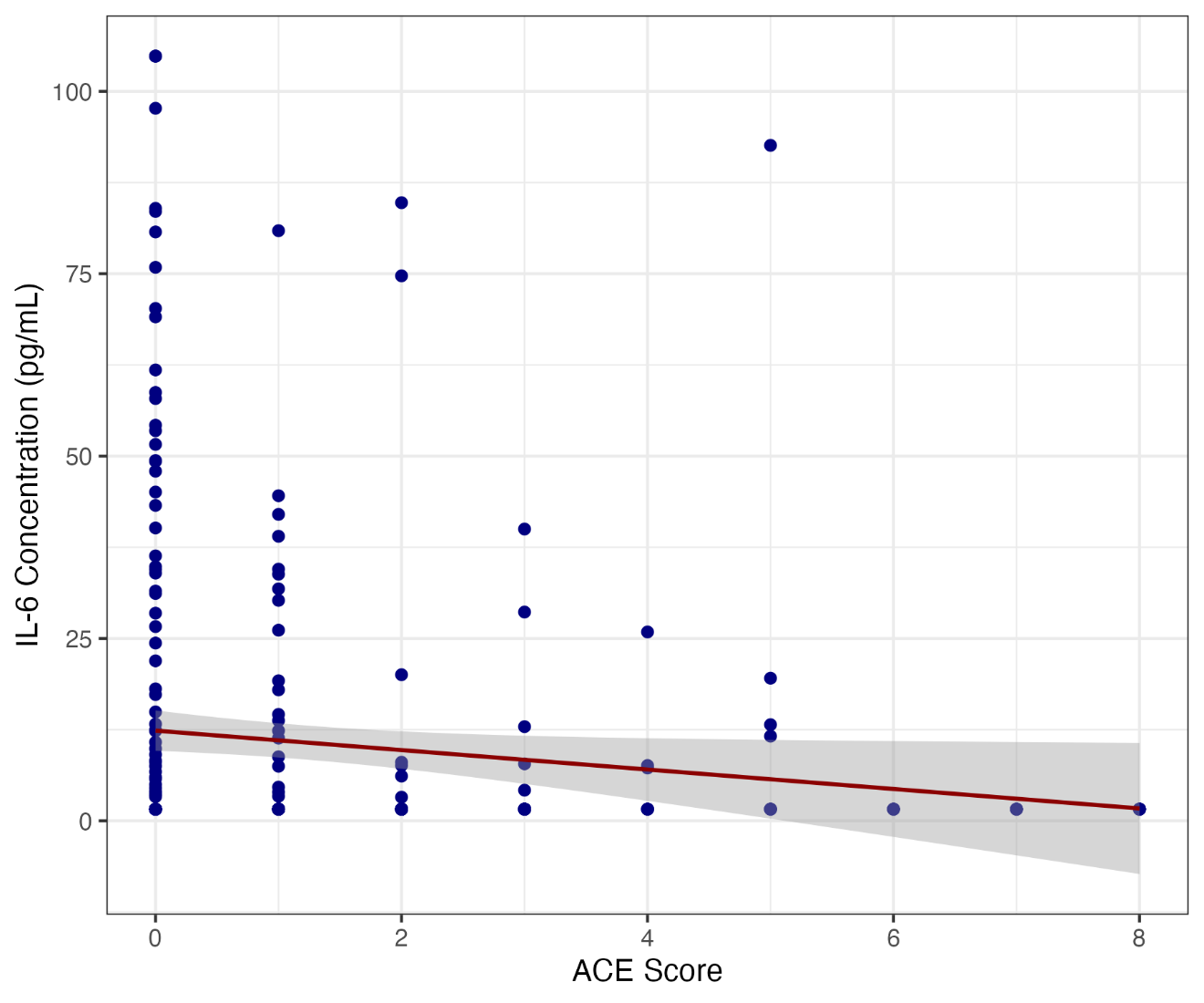
**

**Supplementary Table 1 – Results from tobit regression models assessing relations between cytokine levels and increasingly stratified ACE scores among a sample of pregnant people.**

| Cytokine | Covariate | Adjusted exponentiated β | Interaction model(a)  exponentiated β |
| --- | --- | --- | --- |
|  |  | (*p*-value) | (*p*-value) |
| TNF-α | 2-3 ACEs | 1.006 (0.961) | 0.837 (0.311) |
|  | 4+ ACEs | 1.026 (0.840) | 0.920 (0.698) |
|  | 2-3 ACEs x Financial Stress | -- | 1.025 (0.182) |
|  | 4+ ACEs x Financial Stress | -- | 1.016 (0.494) |
| IL-6 | 2-3 ACEs | 0.411 (0.104) | **0.101 (0.017)** |
|  | 4+ ACEs | 0.368 (0.127) | 0.318 (0.293) |
|  | 2-3 ACEs x Financial Stress | -- | *1.205 (0.059)* |
|  | 4+ ACEs x Financial Stress | -- | 1.031 (0.817) |
| IL-8 | 2-3 ACEs | 0.585 (0.139) | **0.210 (0.015)** |
|  | 4+ ACEs | 0.612 (0.248) | 0.369 (0.192) |
|  | 2-3 ACEs x Financial Stress | -- | **1.142 (0.042)** |
|  | 4+ ACEs x Financial Stress | -- | 1.076 (0.371) |

*Notes: Results generated from six multivariable tobit regression models, one for each cytokine as the outcome variable times two for each model type (adjusted model and adjusted model with interaction term). Multivariable estimates are adjusted for maternal age at delivery, gestational age at blood sample draw, maternal pre-pregnancy BMI, tobacco use during pregnancy, fetal sex, presence of cardiometabolic disorders, and presence of anxiety and/or depression. Reference group is 0-1 ACE for all models. ACE group sample sizes were as follows: 0-1 ACE, n=223; 2-3 ACEs, n=51; 4+ ACEs, n=30). (a) Interaction model refers to multivariable regression models which included an interaction term for categorized ACEs and continuous financial stress.*

**Supplementary Table 2 – Results from ordinal logistic regression models assessing relations between cytokine levels and maternal adversity predictors among a sample of pregnant people.**

| Cytokine(a) | Covariate | Adjusted OR  (*p*-value) | Interaction model(b) OR  (*p*-value) |
| --- | --- | --- | --- |
|  |  |  |  |
| TNF-α | 2+ ACEs | 1.00 (>0.9) | 0.66 (0.30) |
|  | Financial Stress | **1.05 (0.03)** | 1.03 (0.20) |
|  | 2+ ACEs x Financial Stress | -- | 1.06 (0.20) |
| IL-6 | 2+ ACEs | **0.48 (0.03)** | **0.22 (0.004)** |
|  | Financial Stress | 0.96 (0.10) | **0.93 (0.02)** |
|  | 2+ ACEs x Financial Stress | -- | *1.12 (0.06)* |
| IL-8 | 2+ ACEs | 0.67 (0.20) | **0.31 (0.01)** |
|  | Financial Stress | 1.00 (>0.9) | 0.97 (0.3) |
|  | 2+ ACEs x Financial Stress | -- | **1.11 (0.04)** |

*Notes Results generated from six multivariable tobit regression models, one for each cytokine as the outcome variable times two for each model type (adjusted model and adjusted model with interaction term). Multivariable model estimates are adjusted for maternal age at delivery, gestational age at blood sample draw, maternal pre-pregnancy BMI, tobacco use during pregnancy, fetal sex, presence of cardiometabolic disorders, and presence of anxiety and/or depression. (a)* *All cytokine values were split into three categories for modeling. TNF-α was categorized into tertiles based on the distribution (low: < 4.85 pg/mL, medium: 4.85-8.25 pg/mL, high: ≥8.25 pg/mL); IL-6 and IL-8 were categorized into tertiles, undetectable (below LLOQ), below median (excluding undetectable values), and above median (excluding undetectable values). (b) Interaction model refers to multivariable regression models which included at cross product term for categorized ACEs and continuous financial stress.*
